# Antisnake Venom Activity of *Hibiscus aethiopicus* L. against *Echis ocellatus* and *Naja n. nigricollis*

**DOI:** 10.1155/2010/837864

**Published:** 2010-06-06

**Authors:** S. S. Hasson, A. A. Al-Jabri, T. A. Sallam, M. S. Al-Balushi, R. A. A. Mothana

**Affiliations:** ^1^Division of Immunology, Department of Microbiology and Immunology, College of Medicine and Health Sciences, Sultan Qaboos University, P.O. Box 35, Muscat, Oman; ^2^Alex Close, off Gibson Road, Liverpool, L8 1SU, UK; ^3^Department of Community Health, Faculty of Medical Sciences, Al-Baha University, Saudi Arabia; ^4^Pharmacognosy Department, College of Pharmacy, King Saud University, P.O. Box 2457, Riyadh 11451, Saudi Arabia

## Abstract

The objective of the study is to investigate whether the *Hibiscus aethiopicus* L. plant has neutralization activity against venoms of two clinically important snakes. The *H. aethiopicus* was dried and extracted with water. Different assays were performed to evaluate the plant's acute toxicity and its anti-snake venom activities. The results showed that *H. aethiopicus* extract alone had no effect on the viability of C_2_C_12_ muscle cells, but significantly (*P* < .05) protected muscle cells against the toxic effects of *E. ocellatus* venom at 55, 150, and 300 *μ*g/mL. The maximum protective effect of the extract was exhibited at 75 *μ*g/mL. The extract significantly (*P* < .001) inhibited the cytotoxic effects of *E. ocellatus* venom at 300 *μ*g/mL. All rabbits (*n* = 10) and guinea pigs (*n* = 10) were alive after the two weeks of given the lethal dosage 16 g/Kg of the *H. aethiopicus* extract herbal solution. No abnormal behaviour was observed of both groups of animals. All guinea pigs (*n* = 3) treated with venoms alone (5 mg/kg) died. However, all guinea pigs (*n* = 21) treated with venom (5 mg/kg) and the extract (400 to 1000 mg/kg) survived. Guinea pigs (*n* = 3) treated with *Naja n. nigricollis* venom alone (2.5 mg/kg) and guinea pigs (*n* = 21) venom with the extract (400 to 1000 mg/kg) died. The *H. aethiopicus* completely (100%) blocked the haemorrhagic activity of *E. ocellatus* in the egg embryo at 3.3 mg/mL of extract. These findings suggest that *H. aethiopicus* may contain an endogenous inhibitor of venom-induced haemorrhage.

## 1. Introduction

Snake bite remains a public health problem in many countries even though; it is difficult to be precise about the actual number of cases. It is estimated that the true incidence of snake envenomation could exceed 5 million per year [1]. About 100,000 of these develop severe squeal [1]. The global disparity in the epidemiological data reflects variations in health reporting accuracy as well as the diversity of economic and ecological conditions [1]. Accurate records to determine the exact epidemiology or even mortality of snake bite cases are generally unavailable [2]. Hospital records fall far short of the actual number, owing to dependence on traditional healers and practitioners of witchcraft, especially in developing countries. It has been reported that in most developing countries, up to 80% of individuals bitten by snakes first consult traditional practitioners before visiting a medical centre [3, 4]. Owing to the delay, several victims die during transit to the hospital. 

Envenoming by snakes such as *E. ocellatus *and* Naja n. nigricollis* is responsible for several clinical complications of severe systemic and local pathology. For example, *E. ocellatus *leads to inflammation (such as swelling, blistering, and necrosis) and haemorrhages [5] due to both metalloproteases and ecarin (an enzyme that activates prothrombin) [6]. On the other hand envenoming by *Naja n. nigricollis* induced clinical complications different from that caused by *E. ocellatus*. These include local necrosis, haemorrhage, complement depletion [7], and respiratory arrest or paralysis [5, 8]. Moreover, the venom of the *Naja n. nigricollis *consists of phospholipase A_2_ (an anticoagulant enzyme which inhibits the prothrombinase complex by its binding to coagulation factor Xa) [9, 10] and cardiotoxin [11]. Furthermore, in some cases envenoming by *Naja n. nigricollis *can induce corneal ulceration and anterior uveitis [12, 13].

Although an intravenous administration of antivenom, prepared from IgG of venom-immunised horses or sheep, is an effective treatment for systemic envenoming [14], the clinical consensus is that antivenom is of limited effectiveness against the effects of local envenoming that develop rapidly after a bite [15]. Such effects include severe pain, oedema, localized haemorrhage, and necrosis [16] which often results in permanent scarring and deformity. The ineffectiveness of antivenom in treating local envenoming has been attributed to the rapid activity of the toxins and the inability of antivenom IgG to cross the blood/tissue barrier [17, 18]. Despite their smaller size, F(ab_2_)2 and Fab fragments of IgG are also ineffective against the local effects of envenoming, whether administered by intravenous or intramuscular routes [19, 20]. Research to develop a treatment for local envenoming is therefore a clinical priority and has focused on the application of natural [21] or synthetic inhibitors [22] of snake venom potent molecules. 

The use of plant remedies to treat snakebite victims in rural areas and poor communities in the developing countries is a common practice. The natives who are predominantly rural farmers come in contact with snakes during their farming engagements. Due to high cost of hospital treatment and unavailability of antivenoms, most often the rural people find it more convenient to consult native doctors who are acclaimed for curing snakebite patients. Anecdotal evidence abounds to indicate that plant remedies used by the native doctors are effective, and there appears to be a high rate of survival among snakebite patients advanced clinical stages of venom toxicity. 

The present study aims to study the anti-snake venom activities of a local plant, *Hibiscus aethiopicus* L. which is brought over 200 years ago from Africa and regrown in *Bani-Hushash *region, Sana'a Yemen [23]. This plant was found to be used by traditional healers in Bani-Hushash East of Sana'a to treat patients bitten by snakes and/or scorpions [23]. Although* Hibiscus aethiopicus* L. has long been used as a medicinal plant by traditional healers, the validity of the claims made for this plant has not been tested scientifically. In other words *Hibiscus aethiopicus* like many other important plant species, has not been investigated adequately in terms of its anti-snake venom activities. We report in this study and for the first time, how significant this folk tradition medicine is (i.e.,* Hibiscus aethiopicus*) to neutralized snake venom activity, of a typical and highly poisonous snakes such as the West African *E. ocellatus *snake. 

## 2. Materials and Methods

### 2.1. Plant Material

The whole plant of *Hibiscus aethiopicus* was collected with assistance of a traditional healer, from *Bani-Hushaiesh*. Authentication and the taxonomic identification of plant materials was confirmed by Dr. A. Wadieh, Department of Botany, Naser College, in Lahj Governorate, University of Aden, Republic of Yemen. A voucher sample was then deposited in the herbarium of the Department of Botany. One kilogram of the fresh plant was dried under mild sunshine. The dried sample was then pulverized and stored in plastic bags. 

### 2.2. Extraction of Plant Material

The air-dried and pulverized plant material (200 g) was extracted with 250 mL water by using a shaking water-bath at 70°C for 2 hours. The extraction with water was repeated three times. The obtained water extract was filtered and evaporated using a rotary evaporator and freeze dryer to give the crude dried extract. The dried extract was stored at 20°C until used.

### 2.3. Source of Venoms

The venom of *E. ocellatus* was kindly provided by Dr. R. Harrison—the Liverpool School of Tropical Medicine, Liverpool, UK. The venom of *Naja n. nigricollis was *purchased from Sigma Aldrich ltd (Category No. V8377).

### 2.4. Ethics

Ethical approval for this study was obtained from the relevant Ethics and Research Committee of the University of Science and Technology, Sana'a, Yemen. All experiments that involve animals were performed according to ethical standards.

### 2.5. Cytotoxicity Assay

The effects of *Hibiscus aethiopicus* extract and venoms (*E. ocellatus *and* Naja n. nigricollis*) on cultured C_2_C_12_ myoblast cells were investigated separately following the tetrazolium salt (MTT) assay method [24]. Cells were seeded in 96 well microtitre plates (10^4^ cells per well in 100 *μ*L medium) and allowed to attach and reach log phase of growth (24 h). Various concentrations of *Hibiscus aethiopicus* extract (75, 150, and 300 *μ*g/mL) with or without venom (15 *μ*g/mL of *E. ocellatus, *30 *μ*g/mL *Naja n. nigricollis*) were added to each well in 100 *μ*L medium. The cells were incubated the cells at 37°C 18 h. Ten microliters (10 *μ*L) of MTT (5 mg/mL) was added to each well and plates were incubated for 4 h, at 37°C, after which the medium was aspirated from the wells and a volume of 150 *μ*L DMSO was added per well to solubilise the cells. The microplates were shaken for 2 minutes at 400 rpm on a microplate shaker. The optical density was measured at 570 nm using a Dynex MRX plate reader (MTX Lab Systems). The results were statistically analysed using ANOVA and Student's *t*-test.

### 2.6. Antihemorrhagic Test

One-day-old fertile eggs obtained from a local hatchery were incubated till day 4 at 38°C. The eggs were cracked on day 4 into Clingfilm hammocks following a standard method [21, 25] and incubated further till day 6. Discs of 2 mm diameter cut from filter paper (Whatman No. 2) were impregnated with a standard hemorrhagic dose (SHD) of *E. ocellatus *venom (3 *μ*g/1.5 *μ*L) alone or venom and various concentrations (2.5, 5.0, 7.5, and 10 *μ*g/1.5 *μ*L) of *Hibiscus aethiopicus *extract. The discs were placed on the yolk sac membrane over a major bilateral vein and left for 3 h for hemorrhagic corona to form. The corona was measured with a ruler. Control experiments were performed with the buffered saline solution used to prepare the extract and venom solutions. Readings were taken in triplicate. The minimum concentration required to abolish haemorrhage was recorded as the minimum effective neutralizing dose (MEND).

### 2.7. Evaluations of Acute Toxicity of Hibiscus aethiopicus Extract In Vivo Using Both “Oral” and “Intraperitoneal” Routes

Two in vivo assays using both “oral” and “intraperitoneal” routes were performed to evaluate the acute toxicity and the cumulative effect “safety protection” potentialities of the *Hibiscus aethiopicus* composition.

Ethical approval for this study was obtained from the relevant Ethics and Research Committee of University of Science and Technology, Sana'a, Yemen.

#### 2.7.1. Oral Route Acute Toxicity

Two different species of animals rabbits (group-A) and guinea-pigs (group-B), were used in the toxicity profile. Animals were obtained from a well-known animal keeper, Nuccom, Sana'a, Republic of Yemen. Group-A included ten rabbits weighed between 800 g and 1300 g. Group-B included ten guinea-pigs weighed between 300 g and 900 g. Both rabbits and guinea-pigs were given different dosages to investigate the lethal dosage as illustrated in Tables 2 and 3. The extract solution was given orally using anesthesia at variable dosages to reach the optimum of 16 g/kg (a lethal dosage according to the international standardisation for the classification of substances) for each animal group. The animals were observed for behaviour change continuously for a period of two weeks after such administration. Observation was conducted hourly at day 1, and during the following days, observation was conducted 4–6 times per day. Subsequently, blood samples for biochemical assays, alanine aminotransferase (ALT), aspartate aminotransferase (AST), complete blood count (CBC), and gamma glutamyl transpeptidase (GGT) were collected. At the end of the observation period, animals were sacrificed and dissected for adverse effects if any based on histopathology examination of their eyes, liver, lung, and spleen.

#### 2.7.2. Intra-Peritoneal Route Acute Toxicity

Intra-peritoneal (i.p) acute toxicity test was performed instead of the oral route. A total of 35 guinea pigs of both sexes distributed randomly into five groups and being treated i.p. with increasing doses (250, 500, 750, 1000, 1300, and 1600 mg/kg) of *Hibiscus aethiopicus* extract. The fifth group served as control and received an equivalent volume of distilled water. Animals (i.e., guinea pigs) were observed regularly over a period of 24 h for signs of acute toxicity and death.

### 2.8. Evaluations of Hibiscus aethiopicus Extract In Vivo for Anti-Snake Venom Activity

#### 2.8.1. Administration of Both Venom and Extract after Pre Incubation

Seventy eight adult guinea pigs of both sexes (500–600 g) were divided into four groups. Group 1 (of 3 guinea pigs) was injected with *Naja n. nigricollis* venom (2.5 mg/kg) alone. Group 2 was divided into twelve equal subgroups (G2.1–G2.12) of three guinea pigs each. All of the subgroups were injected i.p. with a mixture of *Naja n. nigricollis* venom and *Hibiscus aethiopicus *extract (50, 75, 100, 200, 300, 400, 500, 600, 700, 800, 900, and 1000 mg/kg) accordingly after both venom and extract were incubated in a test tube for 30 minutes. Groups 3 and 4 were similar to Groups 1 and 2 except that *E. ocellatus* venom (5 mg/kg) was used instead of *Naja n. nigricollis.* All animals were observed over the 24 hours. At the end of the observation period, animals were sacrificed and their skins were dissected to examine the neutralisation efficacy of the *Hibiscus aethiopicus* extract.

#### 2.8.2. Administration of Extracts 30 Minutes Prior to Venom Injection

Sixty adult guinea pigs of both sexes (500–600 g) were divided into four equal groups. Group 1 (of 5 guinea pigs) was injected with *Naja n. nigricollis *venom (2.5 mg/kg, i.p.). Group 2 was divided into five equal subgroups (G2.1–G2.5) of five guinea pigs each. All of the subgroups were injected i.p. with the same dose (2.5 mg/kg, i.p.) of *Naja n. nigricollis* venom 30 min after *Hibiscus aethiopicus* extract was administered orally at different concentrations (100, 200, 300, 400, and 1000 mg/kg) by gastric incubation. Groups 3 and 4 were similar to Groups 1 and 2 except that *E. ocellatus* venom (5 mg/kg) was used instead of *Naja n. nigricollis. *All animals were observed over the period of the experiment.

## 3. Results

### 3.1. Cytotoxicity Assay

**Figure 1:**
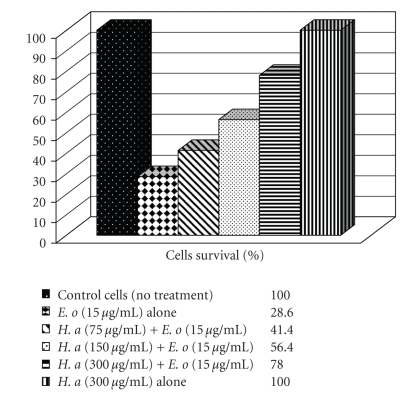
Effects of *Hibiscus aethiopicus* L. extract against the cytotoxic actions of *E. ocellatus* venom on C_2_C_12_ muscle cells. *E. o: Echis ocellatus; H. A: *Extract of *H. aethiopicus* L.

The* Hibiscus aethiopicus* extract alone had no effect on the viability of C_2_C_12_ muscle cells, but it significantly (*P* < .05) protected muscle cells against the toxic effects of * E. ocellatus *venom (30 *μ*g/mL) at all concentrations of the extract tested (55, 150, and 300 *μ*g/mL) (Figure 1). The maximum (67%) protective effect of the extract was exhibited with extract at 75 *μ*g/mL. The extract significantly (*P* < .001) inhibited the cytotoxic effects of *E. ocellatus *venom only at 300 *μ*g/mL (Figure 1). On the other hand the* Hibiscus aethiopicus* extract showed no effect to protect the muscle cells against the toxic effects of *Naja n. nigricollis* (data not shown).

### 3.2. Antihemorrhagic Test

The* Hibiscus aethiopicus* totally (100%) blocked the haemorrhagic activity of* E. ocellatus *in the egg embryo at 5 *μ*g/1.5 *μ*L (3.3 mg/mL) of extract (Table 1). The MEND is 5 *μ*g/1.5 *μ*L.

### 3.3. Evaluations of the Systemic Acute Toxicity of the Hibiscus aethiopicus Extract Using “Oral” Route

All animals (rabbits and guinea pigs) were alive after the two weeks of given the lethal dosage of 16 g/Kg. No abnormal behaviour was observed of both groups of rabbits and guinea-pigs during the observation period. The rabbits and guinea pigs showed normal body weight increase during the two weeks period. Biochemical analysis showed normal range of ALT, AST, CBC, and GGT (Table 2). Inspection of the eyes, liver, lung, and spleen (after scarification and dissection) showed no extraordinary signs. The results when compared to a general acute toxicity index were normal and no acute toxicity was observed.

### 3.4. Evaluations of the Acute Toxicity of the Hibiscus aethiopicus Extract Using “Intra-Peritoneal” Route

Guinea pigs dosed intraperitoneally with *Hibiscus aethiopicus* extract were initially dull with significantly reduced movement for 10–20 minutes. However, neither death nor signs of toxicity were observed even at the highest dose (1600 mg/kg) tested.

### 3.5. Evaluation of Hibiscus aethiopicus Extract In Vivo for Anti-Snake Venom Activity

#### 3.5.1. Administration of Extracts 30 Minutes prior to Venom Injection

All* g*uinea pigs injected with *E. ocellatus* venom (5 mg/kg) alone died. However all guinea pigs injected with both *E. ocellatus* venom and the plant extract (at a concentration of 400 to 1000 mg/kg) survived (Table 3). However, all guinea pigs treated with *Naja n. nigricollis* venom alone (2.5 mg/kg) and/or venom with the water extract (at a concentration of 400 to 1000 mg/kg) died (Table 3). There was no significant difference between the time of death in both the treated and control groups (Table 3).

#### 3.5.2. Administration of Both Venom and Extract after 30 Minutes Pre Incubation

**Figure 2:**
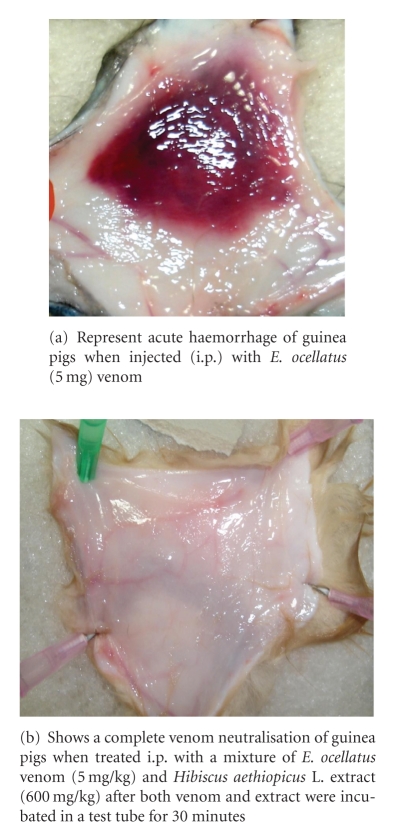
Administration of both venom and extract after 30 minutes pre incubation.

All Guinea pigs treated with venoms *E. ocellatus* 5 mg/kg alone “Group 1” induced acute haemorrhage and died (Figure 2(a)). In contrast all guinea pigs treated with venom and the plant extract “Group 2” at a concentration between 400 and 1000 mg/kg survived and showed no signs of acute haemorrhage (Figure 2(b)). Moreover, all guinea pigs treated with venom and the plant extract below 400 mg/kg died. However, all of the guinea pigs treated with* Naja n. nigricollis* venom 2.5 mg/kg “Group 3” alone and venom with the plant extract “Group 4” died. Although, all guinea pigs treated with a mixture of *Naja n. nigricollis* venom and the extract after preincubation died, the time of death was significantly (*P* < .05) increased from 0.95 h to 5.57 h in the group treated with the mixture of extract and venom in comparison with the control. There was no significant difference between the time of death in the treated and control groups.

## 4. Discussion

Because natural products of higher plants may give a new source of medication, there are many research groups that are now engaged in medicinal plants research not only for the discovery for new drugs but possibly for discovering compounds with novel mechanisms of action that can stimulate new fields of research [23]. Furthermore and considering the high cost of conventional antivenoms and the significant percentage (80%) of patients who react adversely to them [26, 27], a systematic investigation of plant-based remedies for snake bite is justified. Plants used as remedy for snakebite abound in literature [28–31]. However, many of the reported studies lack detailed scientific investigation, which is needed in the development of medicinal agents from plants [28, 30]. Research to develop a treatment for local envenoming is a clinical priority and has focused on the application of natural [21] or synthetic inhibitors [22] of snake venom metalloproteinases (SVMPs) and understanding the pathological role of SVMP-activated proinflammatory cytokines [32–34]. 

In this study we have used one of the main traditional herbal plants called *Hibiscus aethiopicus* which is used as phytotherapy practiced by a large proportion of the Yemen population for the treatment of several clinical complications such as physical, physiological, mental, and social ailments [23] as well as snake envenomation. In comparison to other plants that were adequately investigated worldwide there is little scientific research done to investigate generally the plants of Yemen which are used in herbal medicine. However, no scientific reports were found for *Hibiscus aethiopicus* and its capacity to neutralized snake venom(s). Therefore, this study represents the first report about *Hibiscus aethiopicus* and its uses as antivenom agent. To examine such neutralisation efficacy, two venoms from two different snake species (*E. ocellatus *and* Naja n. nigricollis*) were used. Prior to such examination, the toxicity effect of the *Hibiscus aethiopicus* extract was examined on animals using in vivo assays based on “oral” and “intra-peritoneal” administration routes. 

The results of the oral route when compared to a general acute toxicity index showed normal with no extraordinary syndromes as well as no acute toxicity. However, both routes showed that no death was recorded even at the highest tested dose (16 mg/kg). This was supported further by the biochemical analysis as shown in Table 2. However, results from the intraperitoneally route showed initially dull with significantly reduced movement of the guinea pigs for about 10–20 minutes. In the subsequent experiments we used the *Hibiscus aethiopicus* extract to assess its efficacy to neutralise the haemorrhagic activity of *E. ocellatus* and *Naja n. nigricollis* venoms using an in vivo minimum haemorrhagic dose (MHD) assay utilised to preclinically assess new antivenoms. 

It was interesting to note that results of the evaluation assays of anti-snake venom activity showed that *Hibiscus aethiopicus* induces significant neutralisation capacity against venom of *E. ocellatus* compared to that of *Naja n. nigricollis* venom which showed no antivenom activity at all, as all guinea pigs treated with a preincubation mixture of *Naja n. nigricollis* venom and the extract died. There was no significant difference between the time of death in the treated and control groups. These results were confirmed further by the cytotoxicity assay. 

The *Hibiscus aethiopicus* extract significantly blocked many of the toxic effects of *E. ocellatus* venom in vitro. Remarkably, the extract could not block the neurotoxic activities of *Naja n. nigricollis* (results not shown) venom on chicken blastodermal cells (cbcs) muscle preparation whether the former was added three minutes before or after the venom when the venom's effects on the preparation are normally irreversible by three times washout of the venom. 

Although the component(s) of the plant extract responsible for the antivenom activity observed in the present study has not yet been identified to verify the complete absence or present of tannins, particularly low molecular weight polyphenolic (epi-gallocatechin, epi-catechin etc.) and a long-chain ester of transferulic acid, we presume that it is unlikely that the antivenom activity is due to polyphenolic components. Moreover, in comparison with the antivenom activity of the extract on the *E. ocellatus* venom to that of *Naja n. nigricollis*, it can be observed that it is not possible to be due to the extract acting through a mechanistic intervention rather than a direct physical interaction with the venom in vitro. This is similar to the mode of action of many polyphenolic compounds found in plant extracts. This prospect was confirmed partially by the extract “protective” effects of plant extract when they are preincubated with *E. ocellatus* venom before administration to the biological assay. Therefore, to validate the above speculation(s) future work is necessary to isolate and examine such component(s). 

Another positive attribute of the extract is its antihaemorrhagic activity against *E. ocellatus *venom. We have clearly shown that the extract is very effective against the activity of *E. ocellatus* venom in the circulatory system. These findings reflect that the extract of *Hibiscus aethiopicus* plant may contain an endogenous inhibitor of venom-induced haemorrhage. This obviously would need further investigations.

Despite these protective effects of the plant extract of the *Hibiscus aethiopicus* in vitro, the results obtained from the in vivo experiments were highly encouraging. The extract did protect animals challenged with lethal doses of the *E. ocellatus *venom when the extract and venom were administered independently. Moreover, the extract did not protect animals challenged with lethal doses of the *Naja n. nigricollis* venom when extract and venoms were administered independently. However, haemorrhage induction was significantly reduced (correlated with the increased of the extract concentration) with an increase in the survival time of the guinea pigs treated with a mixture of the extract and the *E. ocellatus* venom after 30 min of preincubation. In addition, none of the guinea pigs treated with a mixture of *Naja n. nigricollis* venom and the extract *Hibiscus aethiopicus* survived. Possibly the plant extract could be effective against viper snake that is, *Naja n. nigricollis,* venom activity in vivo if the experiment model is modified to stimulate actual life experience. In order to obtain more positive results two possible considerations are suggested for future work; first the dose of venom should be adjusted to achieve 75% instead of 100% mortality in the control animal to ensure that they are not challenged with very high dose of venom beyond the dose that any snake can inject into its victim. A very high dose may not give enough time for the extract to induce its antivenom effect. The other suggestion is to give repeated doses of the extract at various time intervals (include an administration of the extract immediately after venom injection), which approximate to what obtains when humans are treated for snakebite. Therefore, this can reflect the therapeutic potential of the extract. Treatment is normally continued until clinical signs of the envenomation disappear.

As venom toxicity within the same species varies and very hard to standardize, the doses that we used in this study to induce lethal effects by both *E. ocellatus *and* Naja n. nigricollis* were previously determined. The concept of the venom toxicity variation is well documented in the literature. For instance, Ode and Asuzu (2006) [35] used different venom concentrations (to that we used) of 10 mg/kg and 6 mg/kg of *E. ocellatus* and *Naja n. nigricollis*, respectively, to induce lethal effects. Moreover, from our previous work with *E. ocellatus* [36–38], for example, we have showed that venom variation occurred both locally and from region to region within the same species. Furthermore, Boche et al. [39] and Broadle [40] reported that snakes of the *Naja n. nigricollis* species have variation in their neurotoxin content both qualitative and quantitative. 

Finally, future works are necessary. First we have to perform a fractionation(s) assay of the *Hibiscus aethiopicus* extract, so that the effective component(s) responsible for the inhibitory effect can be identified. Subsequently, this will allow us to perform the classical toxicity and neutralisation assays including the lethal dose (LD_50_) of the venom as well as to calculate the effective dose (ED_50_) of the extract.

## Figures and Tables

**Table 1:** Effects of *H. aethiopicus* L. extract on the haemorrhage induced by *E. ocellatus* venom in the egg embryo.

Extract/venom	Concentration of extract (*μ*g/1.5 *μ*L)	Haemorrhagic zone (mm)	Reduction from control	*MEND (%)	State of embryo (*μ*g/1.5 *μ*L)
*E. ocellatus* (3 *μ*g/1.5 *μ*L)	—	3.0	—		All died
*H. aethiopicus* L. + * E.o* (3 *μ*g/1.5 *μ*L)	2.5	0.5	85.7		All alive
*H. aethiopicus* L. + * E.o* (3 *μ*g/1.5 *μ*L)	5.0	0.0	100.0	5	All alive
*H. aethiopicus* L. + * E.o* (3 *μ*g/1.5 *μ*L)	10.0	0.0	100.00		All alive

*MEND: Minimum effective neutralising dose; *E.o: Echis ocellatus.*

**Table 2:** Biochemical analysis for acute toxicity of the *Hibiscus aethiopicus* L. plant.

Group (G)	ALT (Mean*)	AST (Mean*)	GGT (Mean*)
G1: Normal (*n* = 4)	**46 **± 4.5*	**90 **± 2.1	**2.2 **± 0.9
G2: Intoxicated control (*n* = 4)	**71 **± 1.8	**193 **± 4.1	**3.4 **± 0.2
G3: Rabbits (*n* = 10)	**50 **± 6.7*	**143 **± 0.8	**5.3 **± 0.1
G4: Guinea pigs (*n* = 10)	**45 **± 3.2	**103 **± 1.3	**3.1 **± 0.7

ALT: alanine aminotransferase;

AST: aspartate aminotransferase;

GGT: gamma glutamyl transpeptidase.

Data are expressed as International Units (IU/mL); *Mean value Significantly different (*P* < .05) compared with respective values (before treatment) using paired Student's *t*-test. *n* = number of animal. *All values presented are means ± SE (standard error). No statistically significant differences were observed.

**Table 3:** Animal status after 30 minutes *prior to venom injection. *

^*¥*^Group	Venom/Extract	*Hibiscus aethiopicus* L. extract (mg /kg)				
		100	200	300	400	1000
1	*Nnn venom *(2.5 mg/kg, i.p.) *alone *	Died	Died	Died	Died	Died
2	*H. Aethiopicus + Nnn *(2.5 mg/kg, i.p.)*	Died	Died	Died	Died	Died
3	*E.o venom* (5 mg/kg) *alone *	Died	Died	Died	Died	Died
4	*H. Aethiopicus + E.o* (5 mg/kg)*	Died	Died	Died	**Alive**	**Alive**

*V *e* *n* *o* *m* *was injected 30 min after Hibiscus aethiopicus L. extract was administered orally by gastric incubation*.

^*¥*^ *Each group has five guinea pigs. Nnn: Naja n. nigricollis; E.o: Echis ocellatus.*
